# Mr. Harup, on Dissolving the Elastic Gum

**Published:** 1807-05

**Authors:** Robert Harrup

**Affiliations:** Chobham


### *To the Editors of the Medical and Physical Journal.*

Gentlemen,

YOUR constant Reader, who is desirous of information respecting the manner of dissolving the elastic gum, will find in the second volume of your Journal, page 83, a very particular account of a method of preparing a solution of that substance in vitriolic æther, and afterwards forming it into tubes. This process, which it wouldbe unnecessary to again give an account of, is extremely simple, and liable to no other objection than that of the expence, as out of sixteen ounces of vitriolic æther, five only are obtained fit for use. Perhaps nitric æther, as it requires no preparation, consequently suffers no waste, may be employed more advantageously; for, according to Lagrange, it is the true solvent of caoutchouc or elastic gum. M. Grossart, by an ingenious method, succeeded in forming this substance into elastic tubes. He cuts a bottle of the gum circularly, in a spiral slip of a few lines in breadth; he then plunges the whole of the slip into vitriolic æther, till it becomes softened; half an hour is generally sufficient for this purpose. The slip is then taken out of the liquid, and one of the extremities is applied to the end of a mould, first rolling it on itself, and pressing it, then mounting spirally along the cylinder, taking care to lay over and compress with the hand every edge, one against the other, so that that there may not be any vacant space, and that all the edges may join exactly; the whole is then to be bound hard with a tape of an inch in width, taking care to turn it the same way with the slip of caoutchouc. Over the tape, packthread is to be applied, in such a manner, that by every turn of the thread joining another, an equal pressure is given to every part. It is then left to dry, and the tube is made. In removing the bandage, great care must be taken that none of the outward surface, which may have lodged within the interstices of the tape, (of which the caoutchouc takes the exact impression) may be pulled asunder. If it is found difficult to withdraw the mould, it may be plunged into hot water. If the mould were previously smoked or rubbed with chalk, it might probably be removed with less difficulty. I should conceive that polished metallic cylinders are the most eligible moulds for this purpose. The same gentleman employed as solvents, also oils of turpentine and lavender, but found that both were much slower of evaporating than æther; and the oil of of turpentine, particularly, appeared always to have a kind of stickiness. Nevertheless, says he, there is a solvent which has not that inconvenience, is cheaper, and may easily be procured by every one; this solvent is *water*. I proceeded, continues he, in the same manner as with æther. The caoutchouc is sufficiently prepared for use when it has been a quarter of an hour in boiling water; by this time its edges are sometimes transparent. It is to be turned spirally round the mould, in the manner described before, and replunged frequently into the boiling water, during the time that is employed in forming the tube. When the whole is bound with packthread, it is to be kept some hours in boiling water, after which it is to be dried, still keeping on the binding. This method may be successfully employed in forming the larger sort of tubes and many other instruments, but I apprehend it would be impracticable to make the small fine tubes in this way. It is much to be wished that a cheap economical method of dissolving caoutchouc could be discovered, so that it might be again formed into whatever shape required, and retain its original qualities. Although many trials have been made, yet still success may be expected by steady perseverance; merely mentioning, in this place, the effects which some substances have on it, may probably save much fruitless labour to any one who is inclined to make such experiments. Sulphuric æther does not dissolve it completely, unless prepared in the manner already alluded to. Diluted nitric acid renders it yellow; concentrated, destroys it. Muriatic acid has no action upon it; it is little affected by alcohol. Oil of lavender, of turpentine, and of spikenard, dissolve it, with the sssistance of a gentle heat; but a mixture of volatile oil and alcohol forms a better solvent for it than oil alone, and the varnish dries sooner. It is said, that if it be boiled in a solution of alum in water, it is rendered softer than in water alone. Yellow wax, in a state of ebullition, may be saturated with it, by putting it, cut in small pieces, gradually into it. By this means a pliable varnish is formed; which may be applied to cloth with a brush, but it still retains a clamminess.

With the wish that the preceding information may be of some service to your correspondent,

I am, &c.

ROBERT HARRUP.

